# Supplementary material for: Constructing ancestral recombination graphs through reinforcement learning
Source: Front Genet. 2025 Apr 29;16:1569358. doi: 10.3389/fgene.2025.1569358 (PMC12069460; doi:10.3389/fgene.2025.1569358)
Supplement: Supplementary file 2 [file DataSheet1.pdf]

frontiers

Supplementary Material

1 SUPPLEMENTARY TABLES AND FIGURES

1.1 Figures

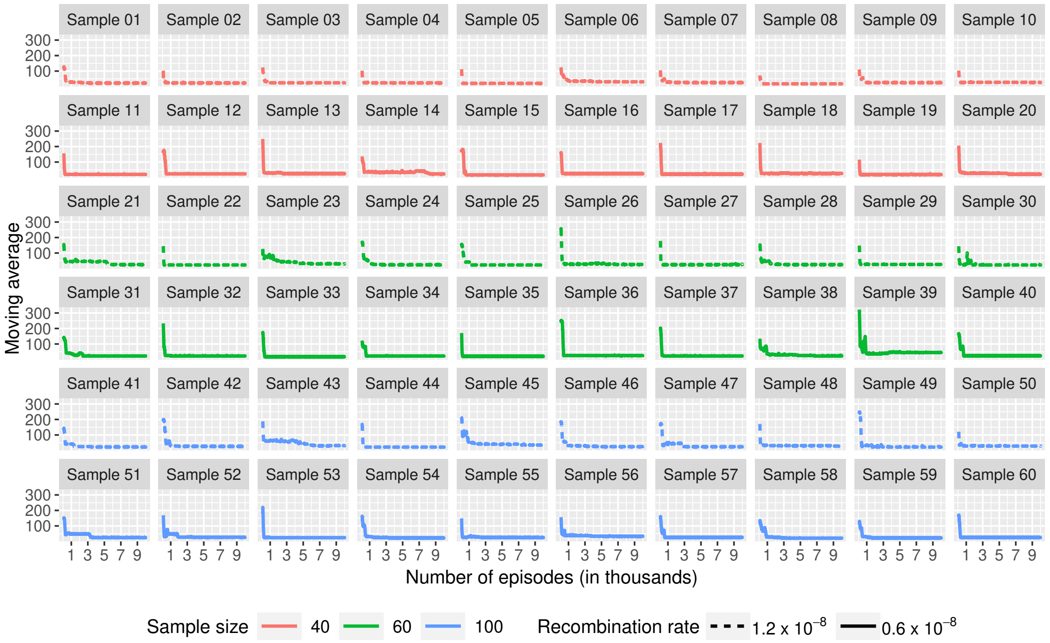

Figure S1. Moving average of the lengths of the ARGs built during training over 100 episodes. Each box represents a learning process using the same sample as initial state. For each sample, we ran 10,000 episodes. 60 samples were used with different sample sizes (40, 60, 100) and different recombination rates (1.2×10^{-8} and 0.6×10^{-8}). The color of the line represents the sample size used and the line type represents the recombination rate.

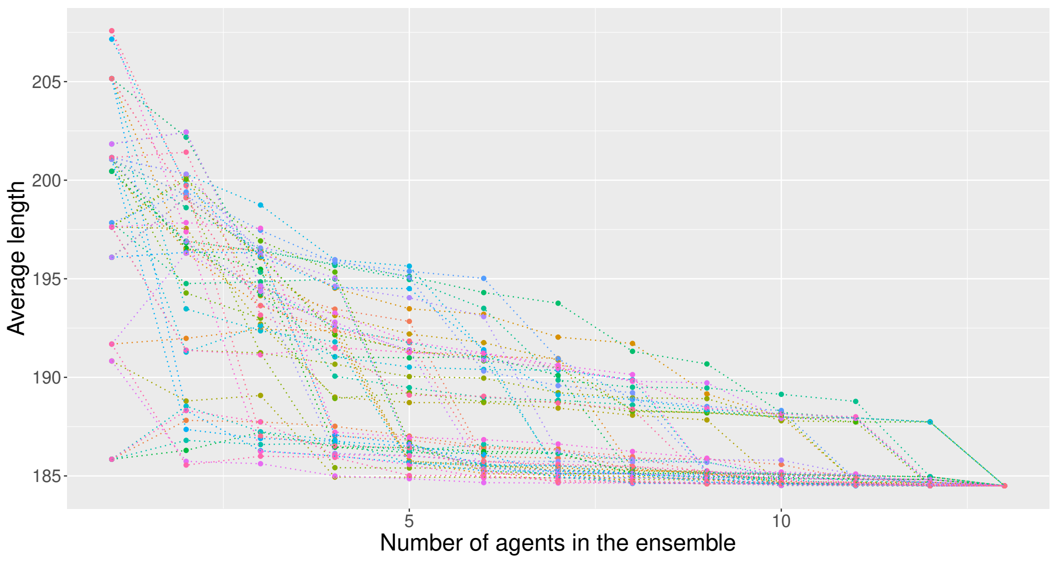

Figure S2. Average length of the ARGs built from 100 test samples of 50 sequences using the third ensemble method (minimum) according to the number of agents used in the ensemble. In the figure, each line shows a different order used to add agents to the ensemble. 50 different orders are shown.

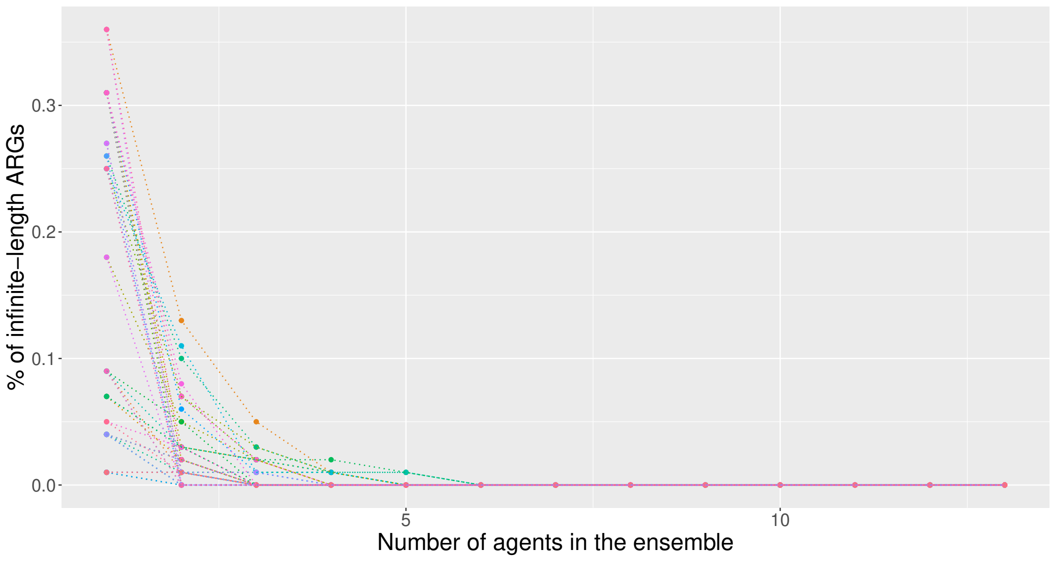

Figure S3. Proportion of infinite-length ARGs built from 100 test samples of 50 sequences using the third ensemble method (minimum) according to the number of agents used in the ensemble. In the figure, each line shows a different order used to add agents to the ensemble. 50 different orders are shown.
